# Visual analysis based on CiteSpace software: a bibliometric study of atrial myxoma

**DOI:** 10.3389/fcvm.2023.1116771

**Published:** 2023-05-12

**Authors:** Ang Gao, Jinghua Yang, Tongru Tian, Yang Wu, Xiaoting Sun, Na Qi, Nan Tian, Xian Wang, Jisheng Wang

**Affiliations:** ^1^ Department of Internal Medicine-Cardiovascular, Dongzhimen Hospital, Beijing University of Chinese Medicine, Beijing, China; ^2^ Department of Gastroenterology, Dongzhimen Hospital, Beijing University of Chinese Medicine, Beijing, China; ^3^ Department of Orthopedics, Zhengzhou Orthopedic Hospital, Zhengzhou, China; ^4^ Department of Encephalology, Dongzhimen Hospital, Beijing University of Chinese Medicine, Beijing, China; ^5^ Department of Andrology, Dongzhimen Hospital, Beijing University of Chinese Medicine, Beijing, China

**Keywords:** bibliometric analysis, atrial myxoma, surgery, citespace, visual analysis

## Abstract

**Objective:**

To use CiteSpace and VOSviewer visual metrology to analyze the research status, frontier hotspots, and trends in research on atrial myxoma.

**Methods:**

The Web of Science core collection database was used to retrieve relevant literature on atrial myxoma from 2001 to 2022. CiteSpace software was used to analyze keywords with a co-occurrence network, co-polymerization class, and burst terms, and a corresponding visual atlas was drawn for analysis.

**Results:**

A total of 893 valid articles were included. The country with the highest number of articles was the United States (*n* = 186). The organization with the highest number of articles was the Mayo Clinic (*n* = 15). The author with the highest number of articles was Yuan SM (*n* = 12). The highest cited author was Reynen K (*n* = 312). The highest cited journal was Annals of Thoracic Surgery (*n* = 1,067). The most frequently cited literature was published in the New England Journal of Medicine in 1995, which was cited 233 times. The keywords co-occurrence, copolymerization analysis, and Burst analysis revealed that the main research focuses were surgical methods, case reports, and genetic and molecular level studies on the pathogenesis of myxoma.

**Conclusions:**

This bibliometric analysis revealed that the main research topics and hotspots in atrial myxoma included surgical methods, case reports, genetic and molecular studies.

## 1. Introduction

In 2015, the International Agency for Research on Cancer (IARC) released the 4th edition of the “WHO Classification of Tumours of the Lung, Pleura, Thymus and Heart” (1), which divided cardiac tumors into benign tumors, tumors of uncertain biologic behavior, germ cell tumors, and malignant tumors. Benign tumors mainly include myxoma, papillary fibroelastoma, and rhabdomyoma. Lipoma (2) and paraganglioma (3) are the rare types of cardiac benign tumor. Cardiac myxoma is the most common tumor of the heart, with an estimated annual incidence of 0.5 per million population (4). Left atrial myxoma is the most common cardiac myxoma, with an incidence of 75%–85% (5, 6). It usually happens to middle-aged people, but it can happen to people of any age. Furthermore, cardiac myxoma usually occurs in women, with a male to female ratio of 1:3 (7, 8).

The tissue origin of atrial myxoma has not been fully defined. At present, it is hypothesized to originate from subendocardial multipotent mesenchymal stem cells near the oval fossa (9). In addition, the pathogenesis of atrial myxoma remains unclear. Ninety percent of atrial myxoma patients rarely relapse after surgical resection. However, certain types of atrial myxoma have a high postoperative recurrence rate, such as the familial cluster onset of Carney syndrome myxoma (10). At present, surgical resection of tumor mass is the best treatment, and there is no effective drug treatment to curb the growth of tumor (11). The current surgical methods used to treat atrial myxoma fall into the following four categories: (1) resection of atrial myxoma by median thoracotomy (12); (2) small incision resection of atrial myxoma (13); (3) thoracoscopic-assisted resection of atrial myxoma (14); and (4) complete endoscopic resection of atrial myxoma (including total thoracoscopic cardiac surgery and robotic cardiac surgery) (15, 16). With rapid developments in the understanding of myxoma at the genetic and molecular level, new targeted drugs may be developed (17, 18); however, some controversy remains. Therefore, it is meaningful to gain knowledge on the development of cardiac myxoma. In this regard, we performed a statistical research analysis of the current literature on cardiac myxoma.

This study aimed to analyze the literature associated with atrial myxoma over 20 years to provide valuable insights into atrial myxoma. We applied CiteSpace and VOSviewer to visually analyze the related literature on atrial myxoma, and discussed its research status and cutting-edge hot research topics, with a view to providing a reference for follow-up research on the development of atrial myxoma.

## 2. Methods

### 2.1. Ethics statement

The present study did not involve any human subjects, and was entirely performed using bibliometric data retrieved from the Web of Science database (WOS, https://www.webofscience.com/wos/woscc/basic-search). Therefore, it is deemed exempt from Institutional Review Board approval.

### 2.2. Data sources and collection

Compared with other databases, Web of Science provides a comprehensive and standardized data set for reference, and has been widely used for bibliometric analysis (19).

The time span was set between January 01, 2001, and October 01, 2022. First, the “WOS Core Collection” was selected on the search page. The search was refined to the subject heading “Atrial Myxoma” and article types “Article” and “Review”. “Plain Text” was chosen for the file format, while “Full Record and Cited References” was chosen for the record content.

The search query string was described as follows: Atrial Myxoma (Topic) and Article or Review Article (Document Types).

### 2.3. Data analysis

The data were downloaded and analyzed by two researchers to assure the accuracy of data and the repeatability of the research. Microsoft Excel 2019 and GraphPad Prism 7 were applied to analyze the targeted files and to export the line charts and tables of the top-cited or productive countries/regions, institutions, authors, journals, references, and keywords.

The 2021 version of the impact factor and journal impact factor quartile, as important indicators to measure the scientific value of research, were also included in the analysis.

### 2.4. Bibliometric analysis and visualization software

CiteSpace (https://citespace.podia.com/download, R6.1.3), a freely available Java-based application, was designed to analyze and visualize trends and patterns in scientific literature, presenting the structure and distribution of scientific knowledge. It focuses on finding critical points in the development of a field or a domain, especially intellectual turning points and pivotal points (20). In this study, CiteSpace was used for keyword clustering and to highlight the results.

VOSviewer (https://www.vosviewer.com/, R1.6.18) is a bibliometric analysis software for mapping knowledge. It can be used for co-word analysis, co-citation analysis, coupling analysis of documents, and to visualize the results (21). In this study, VOSviewer was used to visualize countries, authors, institutional collaborations, cited journals, keyword co-occurrences, and to construct density maps.

## 3. Results

### 3.1. Analysis of global publishing trends

**Figure 1:**
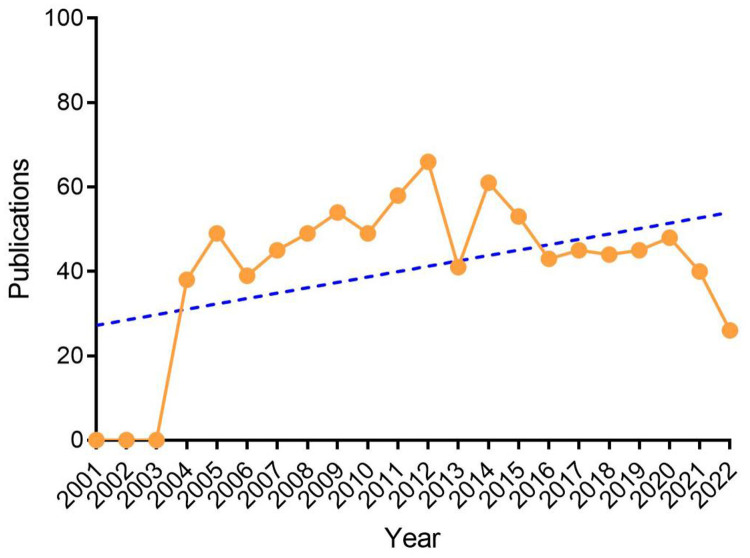
Annual trends of global publications.

A total of 1,430 relevant documents were retrieved; 537 were excluded due to being conference summaries, letters, news, etc., and 893 valid documents were finally included. Since the establishment of the database, there are few early studies in this field. From 2001 to 2003, no original articles or reviews matching the search term “Atrial Myxoma” were included. From 2004 to 2021, the average annual number of papers reported each year was 48, with the highest number in 2012 being 66. Although atrial myxoma is an uncommon disease in the field of cardiology, certain scholars have studied it every year in recent decades, with a steady trend increase over time; however, after 2019, the number of documents issued declined, which may be related to the COVID-19 pandemic (Figure 1).

### 3.2. Analysis of distribution and co-operation of leading countries/regions

**Figure 2:**
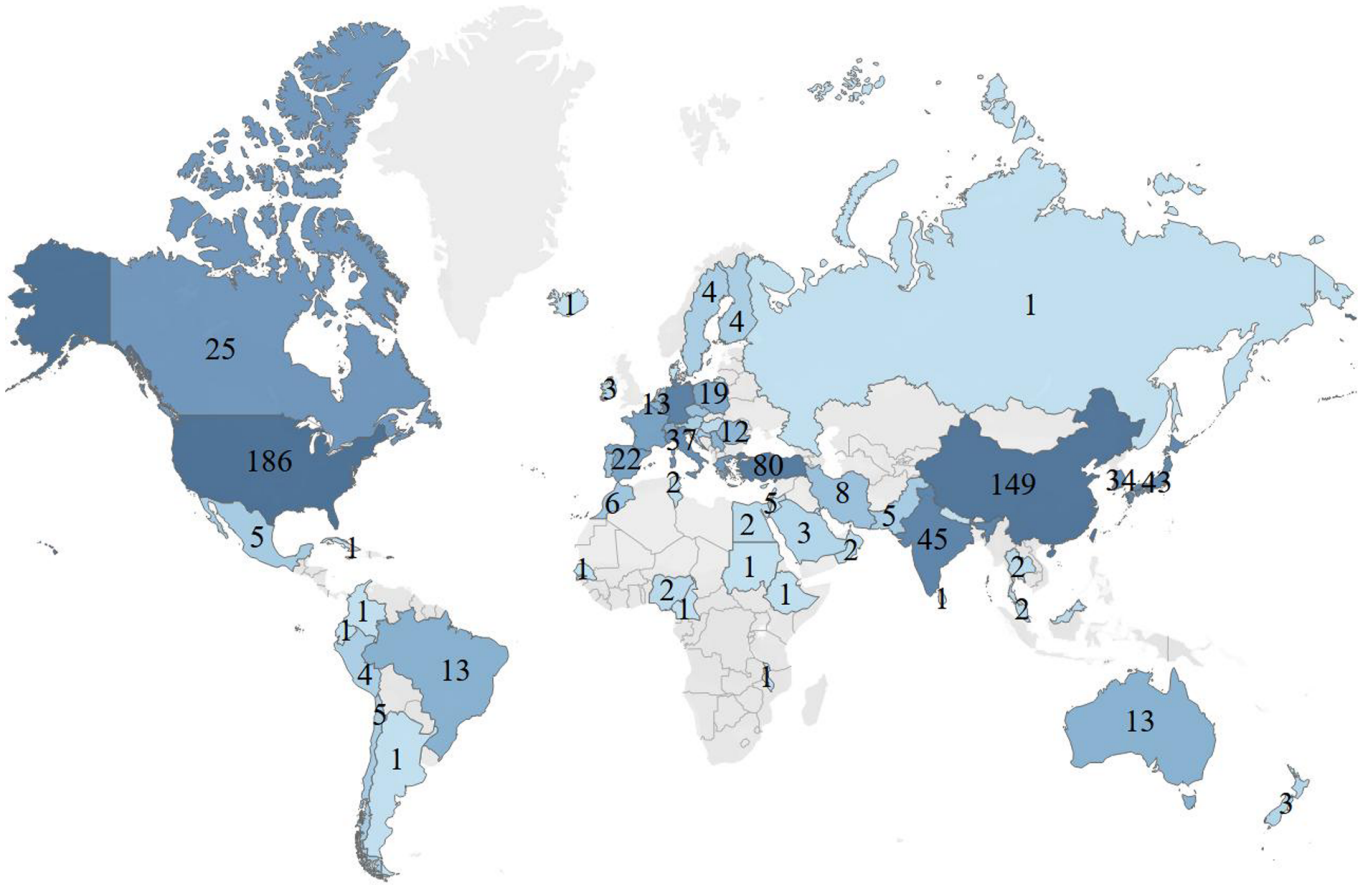
Global analysis of the research trends in atrial myxoma based on the origin of the publications.

A total of 48 countries/regions have published papers on atrial myxoma. The United States of America (USA) had the highest number of publications (*n* = 186, 20.83%), followed by China (*n* = 136, 15.23%), Turkey (*n* = 80, 8.96%), Germany (*n* = 65, 7.28%), United Kingdom (*n* = 45, 5.04%), India (*n* = 45, 5.04%), Japan (*n* = 43, 4.82%), Italy (*n* = 37, 4.14%), South Korea (*n* = 34, 3.81%), and Canada (*n* = 25, 2.80%) (Table 1 and Figure 2).

**Table 1:** Top 10 countries/regions by publications and citations.

Rank	Countries/Regions	Publications	% (of 893)	Rank	Countries/Regions	Citations
1	USA	186	20.83	1	USA	2,392
2	China	136	15.23	2	Germany	1,084
3	Turkey	80	8.96	3	China	1,027
4	Germany	65	7.28	4	England	750
5	England	45	5.04	5	Italy	497
6	India	45	5.04	6	Turkey	482
7	Japan	43	4.82	7	Japan	379
8	Italy	37	4.14	8	Canada	373
9	South Korea	34	3.81	9	Switzerland	353
10	Canada	25	2.80	10	India	329

**Figure 3:**
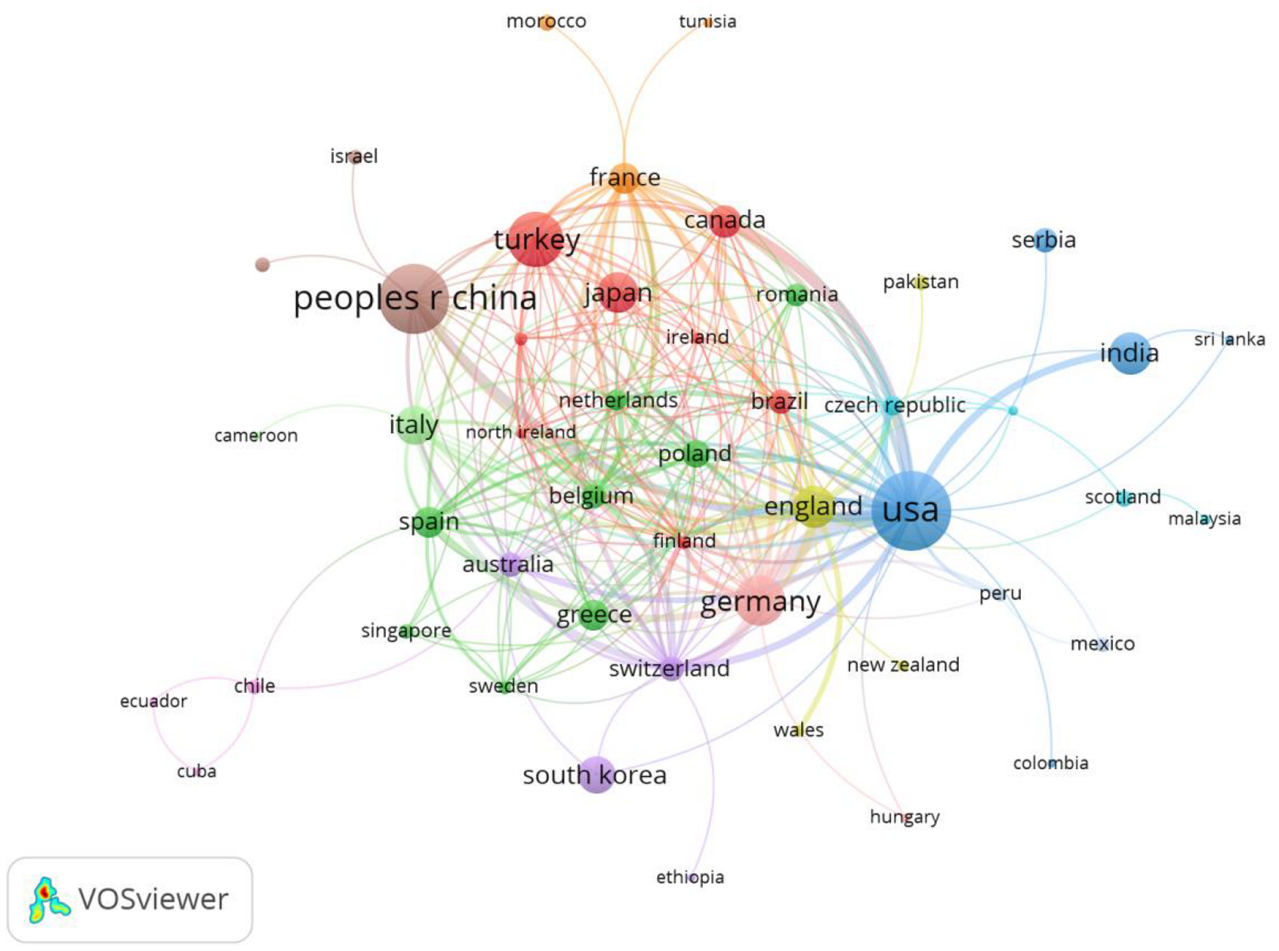
Co-occurrence map of countries/regions. The size of the nodes represents the number of articles; the thickness of the curve represents the strength of the collaboration; the colors represent different collaboration groups.

The highest number of total citations were from the USA (2,392), followed by Germany (*n* = 1,084), China (*n* = 1,027), United Kingdom (*n* = 750), Italy (*n* = 497), Turkey (*n* = 482), Japan (*n* = 379), Canada (*n* = 373), Switzerland (*n* = 353), and India (*n* = 329) (Table 1). Therefore, these countries were shown to be interested in atrial myxoma-related research. VOSviewer was used to analyze the co-operation between different countries. The line between nodes indicates co-authorship between countries; the thicker the line, the stronger the co-operative relationship. The results showed that the USA, China, and Turkey had the highest levels of co-operation with other countries; however, co-operation between the other countries showed a weak relationship (Figure 3).

### 3.3. Analysis of distribution and co-operation of leading institutions

A total of 23 institutions were involved in publishing atrial myxoma-related papers. The top five institutions with the highest number of publications were the Mayo Clinic (*n* = 15), Fujian Medical University (*n* = 13), Zhejiang University (*n* = 9), All India Institute of Medical Sciences (*n* = 9), and Sichuan University (*n* = 8) (Table 2).

**Table 2:** Top 10 institutions by publications and citations.

Rank	Organizations	Publications	Original country	Rank	Organizations	Citations	Original country
1	MayoClinic	15	USA	1	MayoClinic	474	USA
2	Fujian Medical University	13	China	2	Washington University	170	USA
3	Zhejiang University	9	China	3	University of Chicago	167	USA
4	All India Institute of Medical Sciences	9	India	4	University Hospitals Leuven	152	Belgium
5	Sichuan University	8	China	5	Yokohama City University	149	Japan
6	Capital Medical University	6	China	6	Dresden University of Technology	139	Germany
7	Shandong University	6	China	7	University of Alberta	128	Canada
8	Harvard University	6	USA	8	Harvard University	125	USA
9	University of Toronto	5	Canada	9	All India Institute of Medical Sciences	122	India
10	University Hospitals Leuven	5	Belgium	10	Univ London Imperial Coll Sci Technol Med	114	England

**Figure 4:**
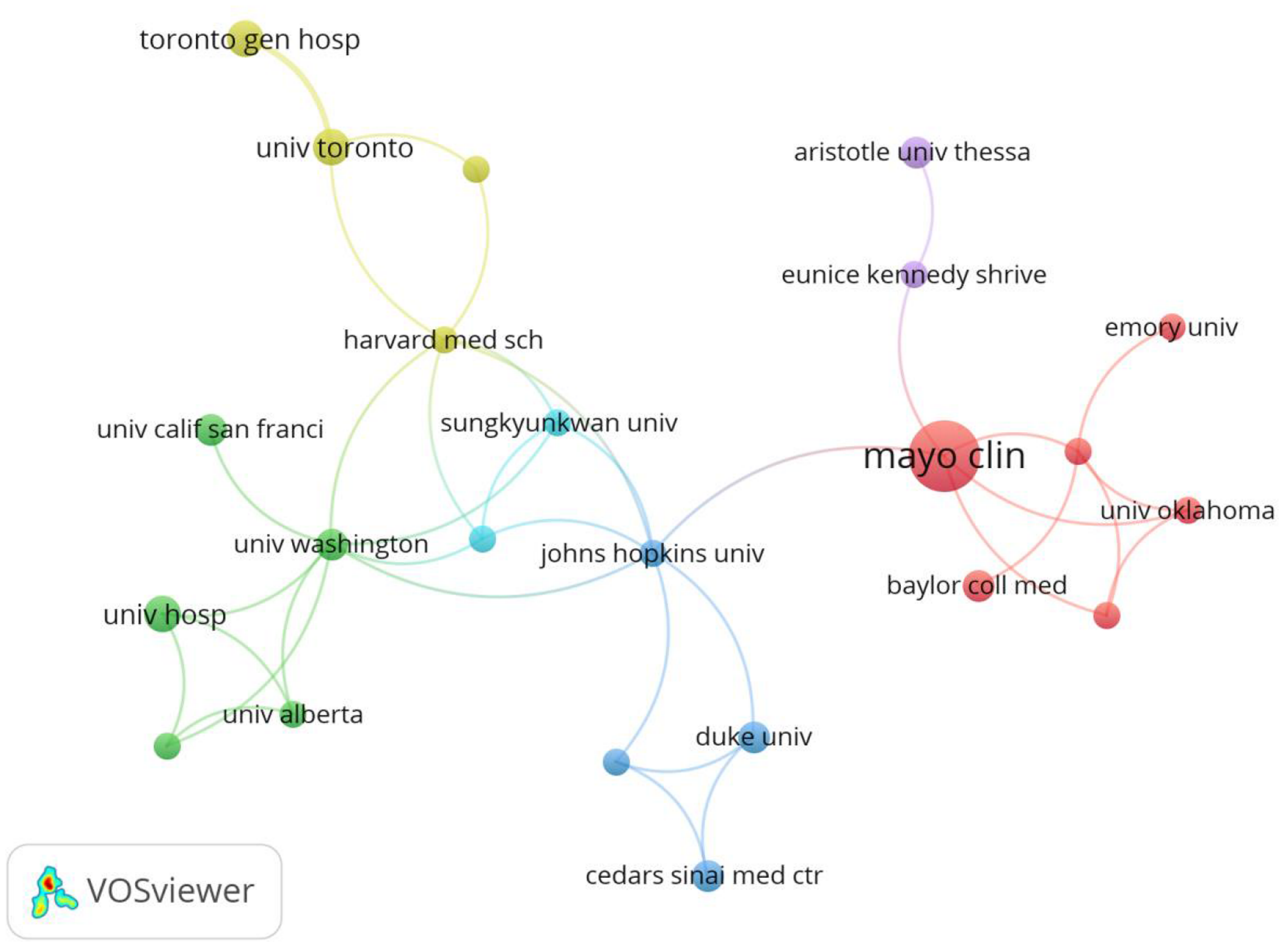
Co-occurrence map of institutions. The size of the nodes represents the number of articles; the thickness of the curve represents the strength of the collaboration; the colors represent different collaboration groups.

The top five institutions with the highest number of total citations were the Mayo Clinic (*n* = 474), Washington University (*n* = 170), University of Chicago (*n* = 167), University Hospitals Leuven (*n* = 152), and Yokohama City University (*n* = 149) (Table 2). A co-operation network has been formed among various institutions; however, the co-operation is relatively decentralized and limited, without close co-operation. Most institutions with a high volume of publications and high number of citations are from the USA and China, thus, research in other countries needs to be improved. The phenomenon of a high number of papers issued by institutions in the USA and China may be related to the incidence rate and more scientific research funding (Figure 4).

### 3.4. Analysis of authors and co-cited authors

**Figure 5:**
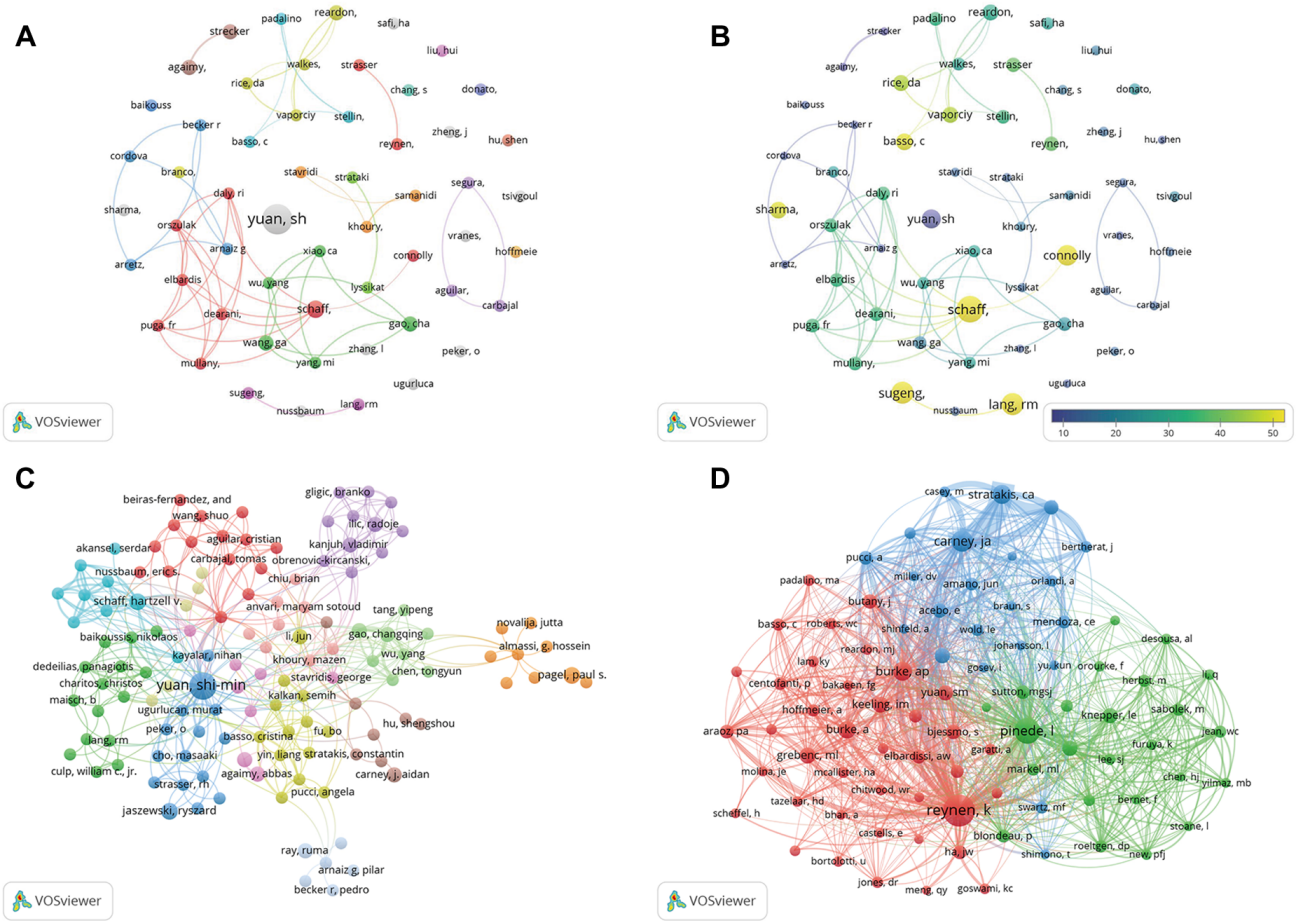
(**A**) Co-occurrence map of authors. The size of the nodes represents the number of articles. (**B**) Superimposition map of cited authors. The size of the nodes represents the number of articles. (**C**) Cited authors analysis map. The size of the nodes represents the number of citations. (**D**) Co-cited authors analysis map. The size of the nodes represents the number of co-citations.

The author co-occurrence analysis identified the core authors in the atrial myxoma-related research field and the strength of collaboration between authors. Regarding the co-cited analysis, when two authors or papers are cited by a third author or paper at the same time, the two authors or papers have a co-cited relationship. Among the authors, Yuan SM (*n* = 12), Schaff HV (*n* = 4), Reardon M (*n* = 3), Gao C (*n* = 3), Wang G (*n* = 3), Agaimy A (*n* = 3), and Strecker T (*n* = 3) had the highest number of publications (Table 3 and Figure 5A). Yuan SM, the author with the highest number of articles, has performed in depth studies on atrial myxoma; however, the author is relatively isolated and has little co-operation with other authors. A co-operation network has been formed among scholars, but it is relatively decentralized (Figure 5A). The closer the color of the nodes are to yellow in Figure 5B, the more authors are cited. The citation analysis showed that Schaff HV (*n* = 241), Lang R (*n* = 167), Sugeng I (*n* = 167), Connolly H (*n* = 147), and Yuan SM (*n* = 127) had the most citations. Schaff HV is active and influential in this research field (Table 3, Figures 5B,C). The co-citation analysis showed that Reynen K (*n* = 312), Pinede I (*n* = 195), Carney J (*n* = 129), Burke A (*n* = 92), and Stratakis C (*n* = 92) had the most co-citations (Table 3 and Figure 5D).

**Table 3:** Top 10 authors and co-cited authors.

Rank	Authors	Publications	Rank	Authors	Citations	Rank	Co-cited authors	Co-citations
1	Yuan SM	12	1	Schaff HV	241	1	Reynen K	312
2	Schaff HV	4	2	Lang R	167	2	Pinede I	195
3	Reardon M	3	3	Sugeng I	167	3	Carney J	129
4	Gao C	3	4	Connolly H	147	4	Burke A	92
5	Wang G	3	5	Yuan SM	127	5	Stratakis C	92
6	Agaimy A	3	6	Basso C	97	6	Burke A	80
7	Strecker T	3	7	Sharma S	96	7	Grebenc M	66
8	Lang R	2	8	Reardon M	89	8	Keeling I	64
9	Sugeng I	2	9	Rice D	89	9	Yuan SM	60
10	Connolly H	2	10	Vaporciyan A	89	10	Lee VH	58

### 3.5. Analysis of keyword co-occurrence, clustering, and burst terms

**Figure 6:**
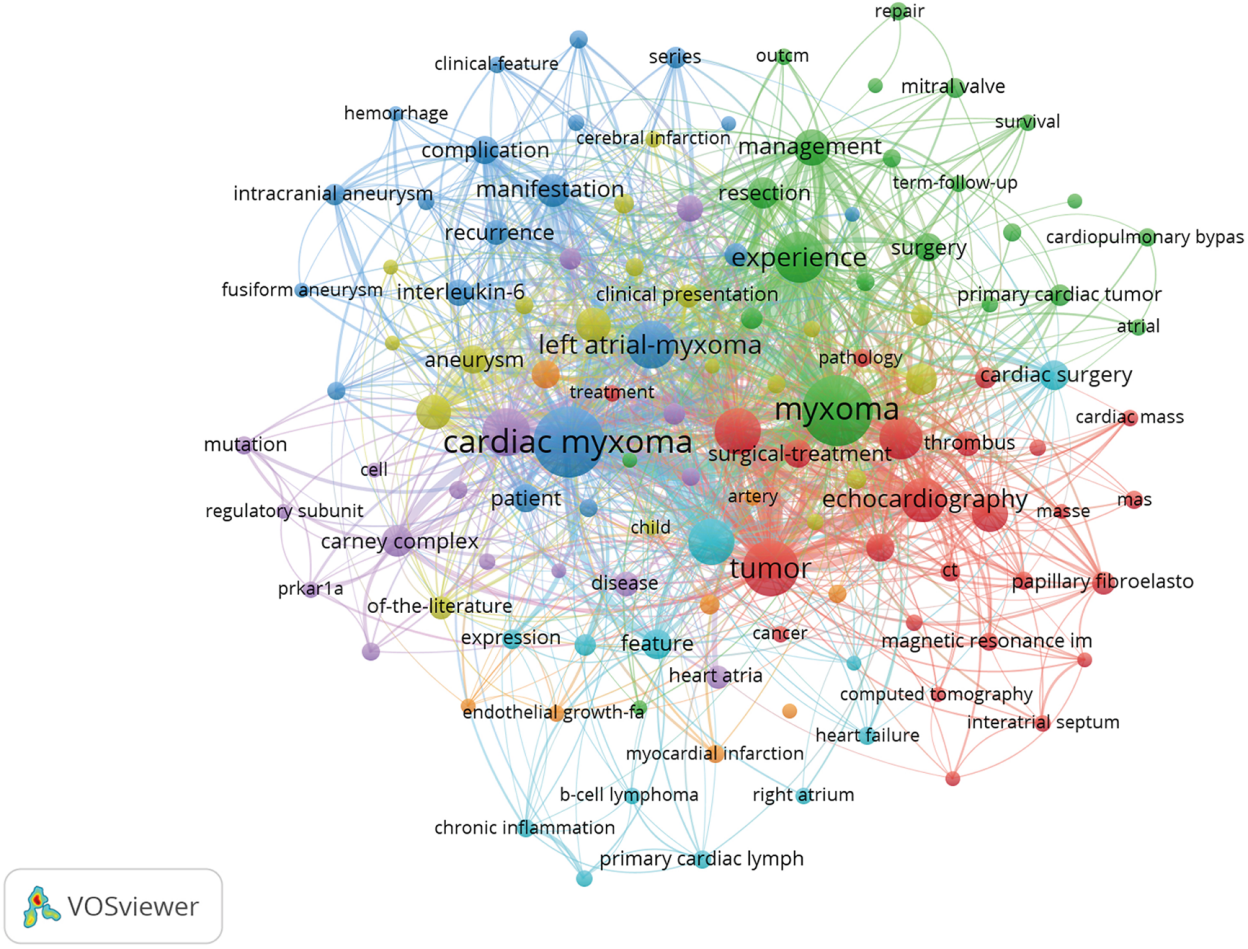
Keyword co-occurrence analysis map obtained using VOSviewer. The size of the nodes represents the number of occurrences; the thickness of the curve represents the strength of collaboration; the different colors represent the different clusters.

**Figure 7:**
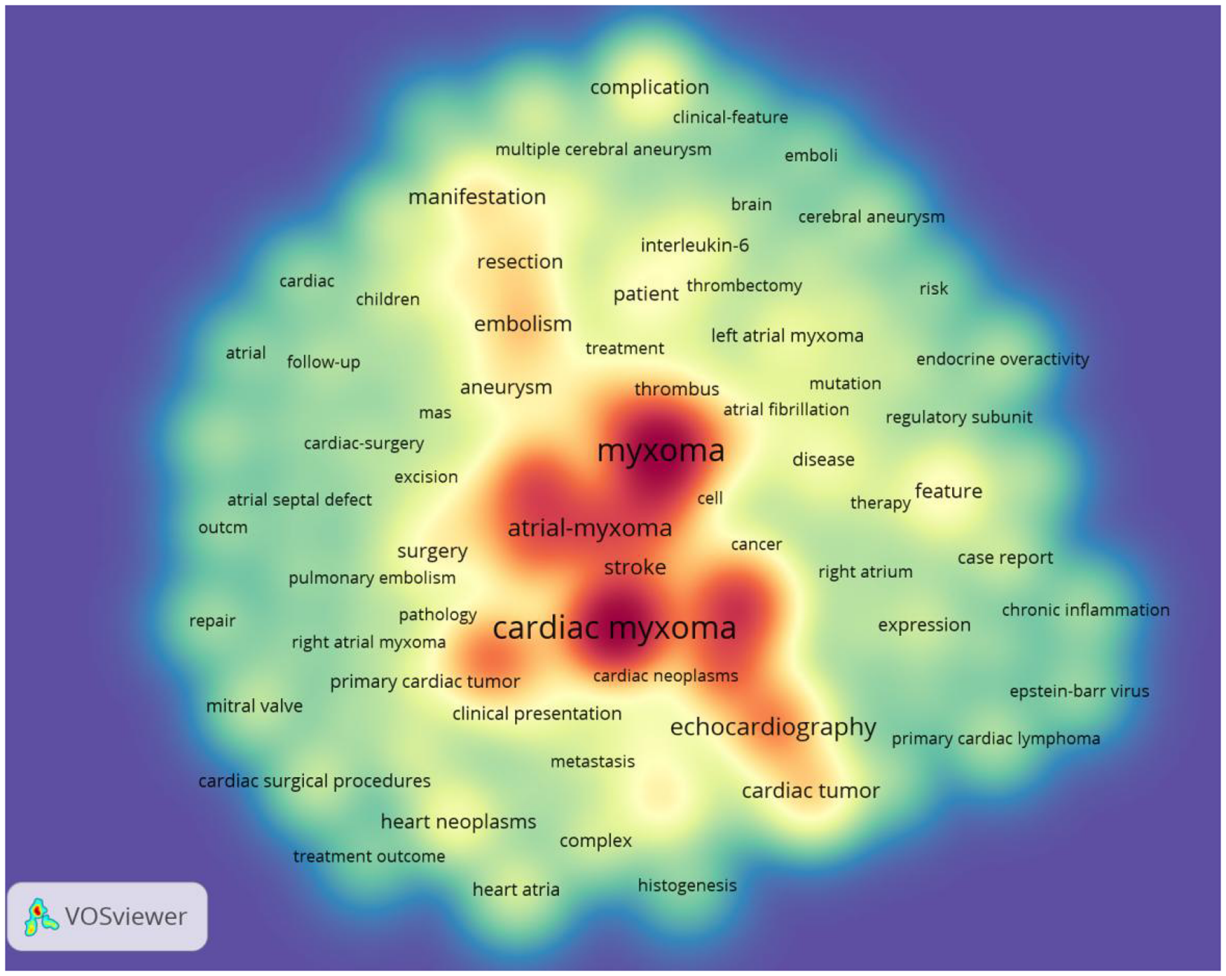
Keyword density visualization analysis. The higher the intensity of the red color node, the higher the number of keywords.

**Figure 8:**
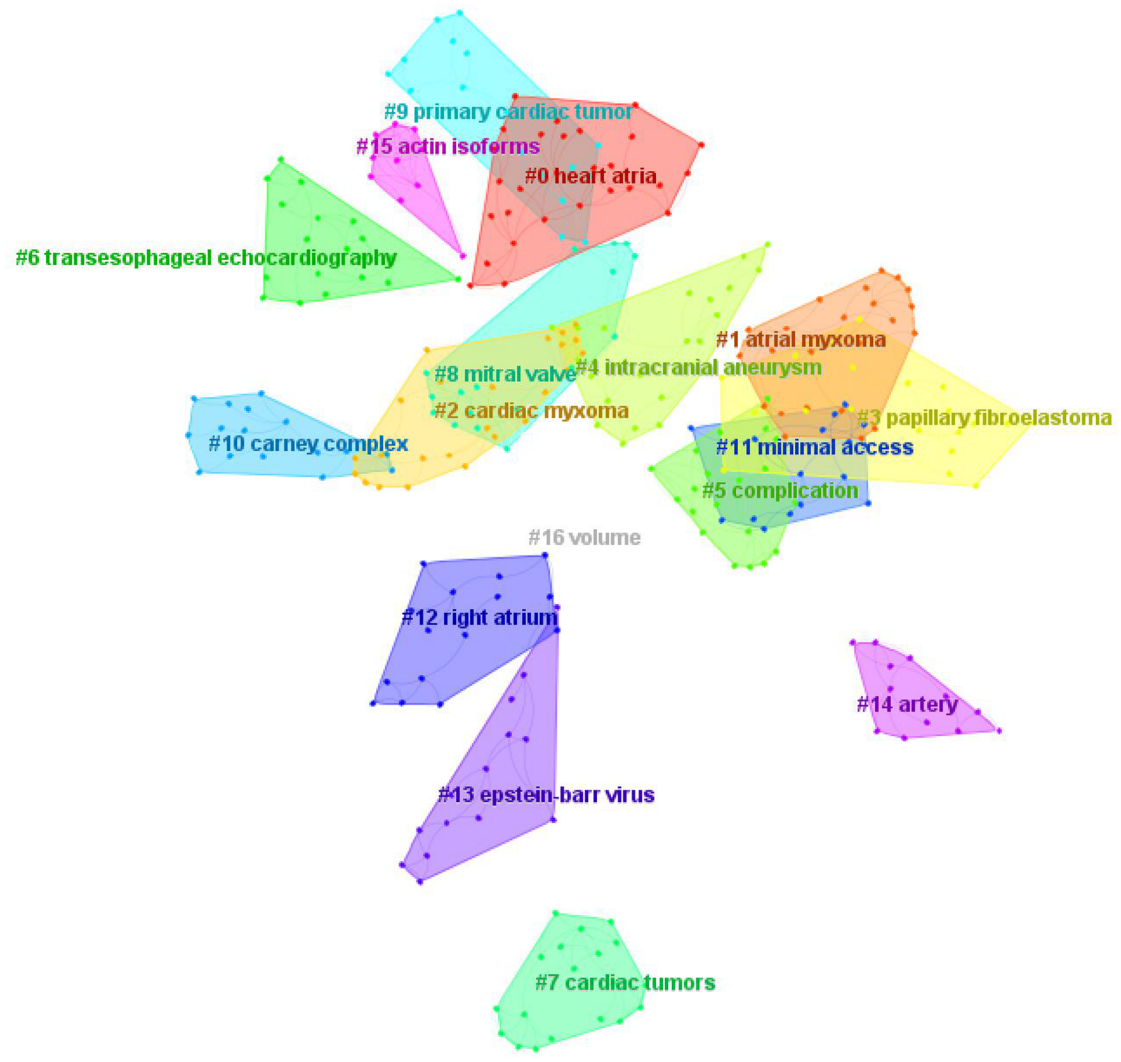
Keyword clustering map analysis through CiteSpace. A total of 17 categories of keywords were obtained. The different color blocks represent different keyword clusters.

**Figure 9:**
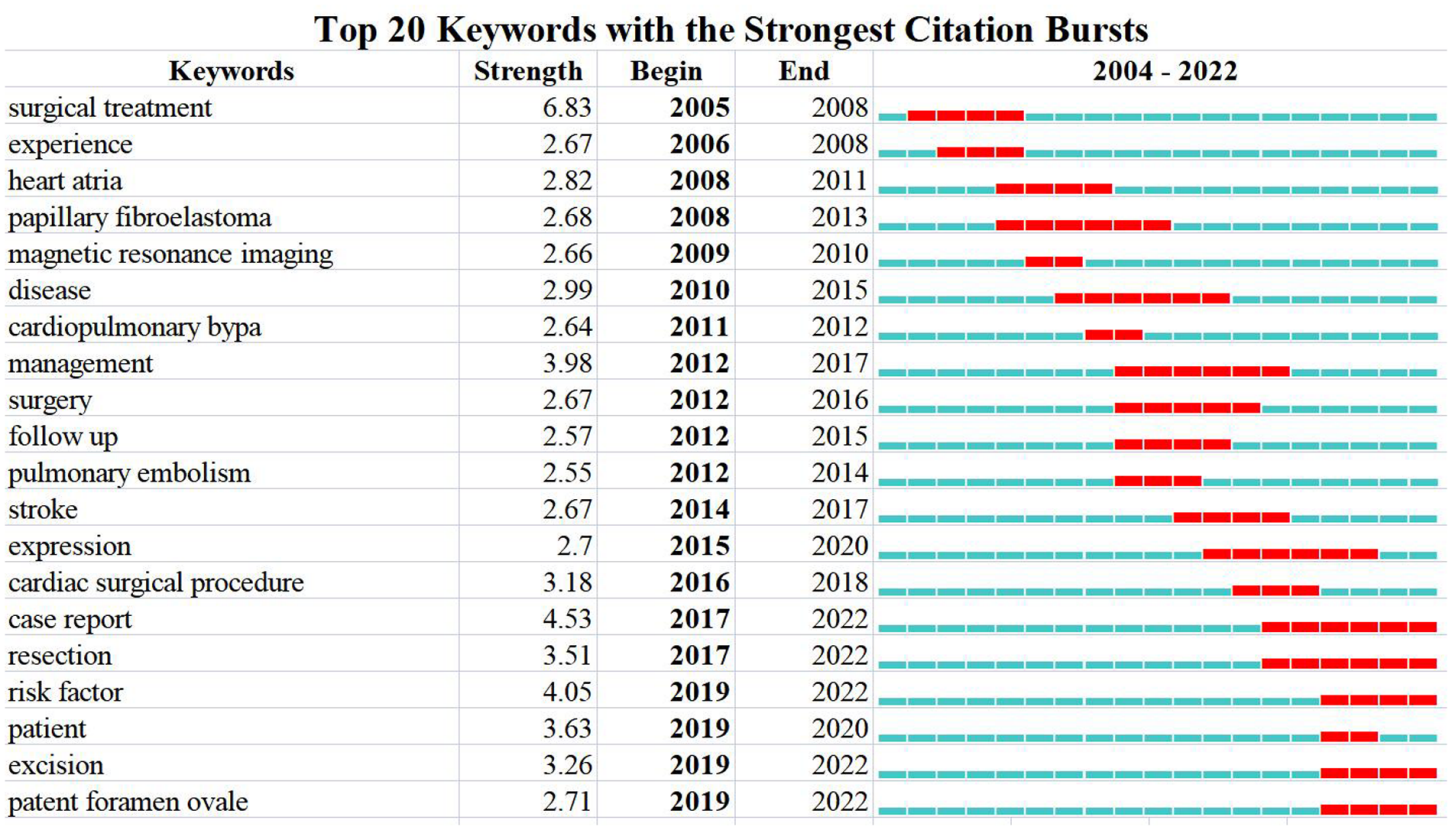
Keywords burst analysis by CiteSpace.

Through keyword co-occurrence and prominence analysis, we can identify the changing trend of research topics over time. A total of 122 keywords were obtained; the top 10 keywords were cardiac myxoma (*n* = 268), myxoma (*n* = 261), tumor (*n* = 153), experience (*n* = 122), left atrial-myxoma (*n* = 104), atrial-myxoma (*n* = 104), diagnosis (*n* = 103), atrial myxoma (*n* = 100), echocardiography (*n* = 91), and heart (*n* = 84) (Figures 6, 7). By rerunning the software, selecting the pathfinder in the clipping mode, and using the logarithmic likelihood algorithm (LLR) to extract the nominal terms from the keywords in the cited literature to name the clusters, a total of 17 clustering words were obtained as follows: #0 heart atria, #1 atrial myxoma, #2 cardiac myxoma, #3 papillary fibroelastoma, #4 intracranial aneurysm, #5 complication, #6 transesophageal echocardiography, #7 cardiac tumors, #8 mitral valve, #9 primary cardiac tumor, #10 Carney complex, #11 minimal access, #12 right atrium, #13 Epstein–Barr virus, #14 artery, #15 actin isoforms, #16 volume (Figure 8). In addition, a total of 20 keyword-highlighted analysis results were obtained. From 2008 to 2012, the highlighted keywords were surgical treatment, experience, heart atria, papillary fibroelastoma, magnetic resonance imaging, disease, and cardiopulmonary bypass. From 2013 to 2017, the highlighted keywords were management, surgery, follow-up, pulmonary embolism, stroke, expression, and cardiac surgical procedure. Furthermore, from 2018 to 2022, the highlighted keywords were case report, resection, risk factor, patient, excision, patent foramen ovale (Figure 9).

### 3.6. Analysis of leading journals and co-cited journals

The top five high-yield journals were the Journal of Cardiac Surgery (*n* = 27), Texas Heart Institute Journal (*n* = 27), Heart Surgery Forum (*n* = 22), Echocardiography—A Journal of Cardiovascular Ultrasound and Allied Techniques (*n* = 22), Journal of Cardiothoracic Surgery (*n* = 21), and the Journal of Cardiothoracic and Vascular Anesthesia (*n* = 21). The most cited journals were Annals of Thoracic Surgery (*n* = 284), Journal of Cardiac Surgery (*n* = 243), Texas Heart Institute Journal (*n* = 238), Journal of Cardiothoracic Surgery (*n* = 199), and the International Journal of Cardiology (*n* = 190). The division of cited journals is mostly Q4, which indicates that the overall level of research on atrial myxoma require further improvement (Table 4).

**Table 4:** Top 10 journals by publications and citations.

Rank	Journals	Publications	IF (2023)	JIF quartile
1	Journal of Cardiac Surgery	27	1.62	Q3
2	Texas Heart Institute Journal	27	0.77	Q4
3	Heart Surgery Forum	22	0.60	Q4
4	Echocardiography—A Journal of Cardiovascular Ultrasound and Allied Techniques	22	1.50	Q4
5	Journal of Cardiothoracic Surgery	21	1.51	Q4
6	Journal of Cardiothoracic and Vascular Anesthesia	21	2.68	Q3
7	Annals of Thoracic Surgery	18	4.28	Q2
8	Cardiovascular Pathology	16	3.67	Q2
9	Medicine	16	1.51	Q3
10	Journal of Thoracic and Cardiovascular Surgery	15	4.99	Q1
Rank	Journals	Citations	IF (2021)	JIF quartile
1	Annals of Thoracic Surgery	284	4.28	Q2
2	Journal of Cardiac Surgery	243	1.62	Q3
3	Texas Heart Institute Journal	238	0.77	Q4
4	Journal of Cardiothoracic Surgery	199	1.51	Q4
5	International Journal of Cardiology	190	3.34	Q2
6	Echocardiography—A Journal of Cardiovascular Ultrasound and Allied Techniques	175	1.50	Q4
7	Cardiovascular Pathology	161	3.67	Q2
8	European Journal of Cardio-thoracic Surgery	150	3.96	Q1
9	Interactive Cardiovascular and Thoracic Surgery	118	1.978	Q3
10	Interventional Neuroradiology	111	1.64	Q4

**Figure 10:**
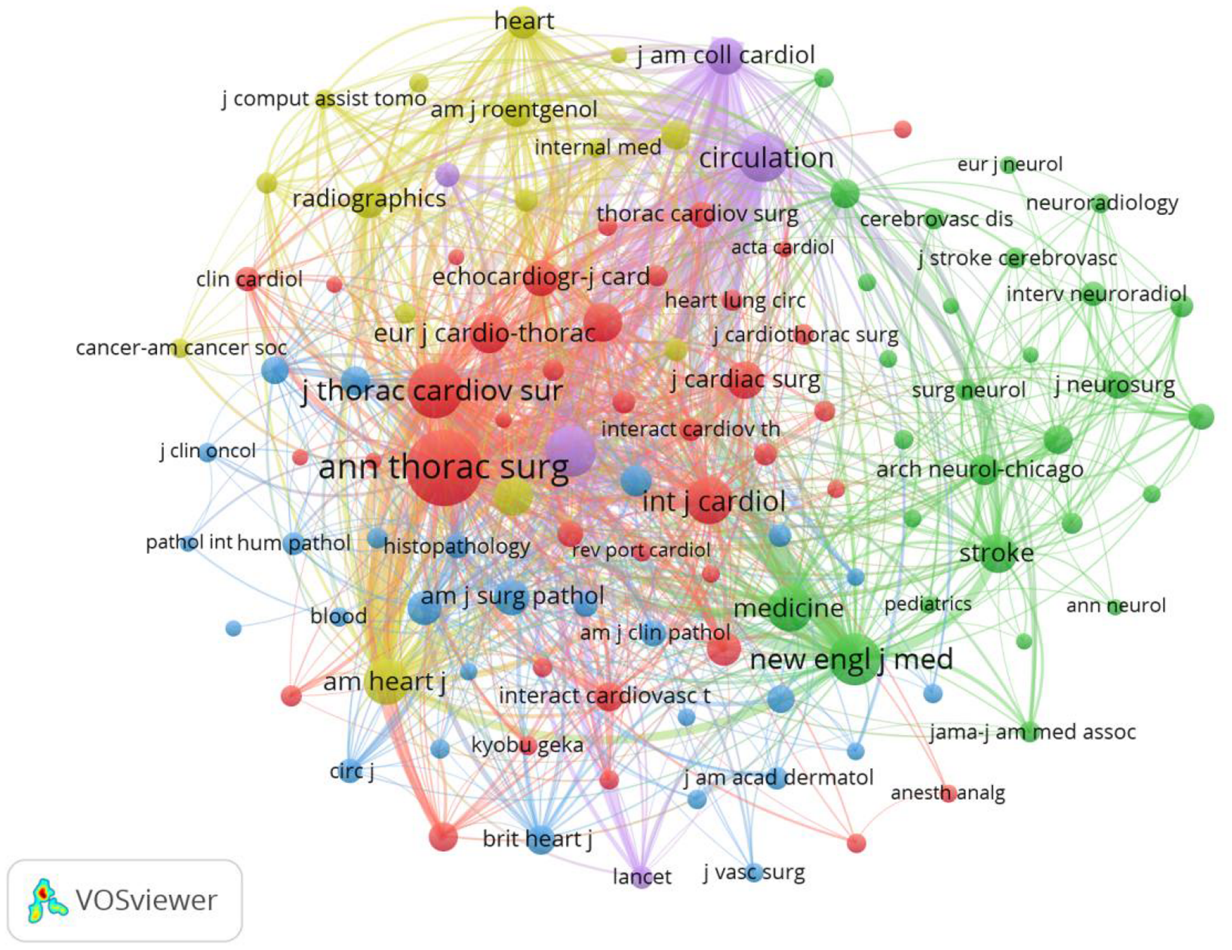
Co-cited journals analysis map. The size of the nodes represents the number of co-citations.

Analysis of the co-cited journals showed that 113 journals were co-cited. The top five co-cited journals were Annals of Thoracic Surgery (*n* = 1,067), Journal of Thoracic and Cardiovascular Surgery (*n* = 517), New England Journal of Medicine (*n* = 454), American Journal of Cardiology (*n* = 414), and Circulation (*n* = 409) (Table 5 and Figure 10).

**Table 5:** Top 10 co-cited journals.

Rank	Co-cited journals	Co-citations	IF (2023)	JIF quartile
1	Annals of Thoracic Surgery	1,067	4.28	Q2
2	Journal of Thoracic and Cardiovascular Surgery	517	4.99	Q1
3	New England Journal of Medicine	454	154.07	Q1
4	American Journal of Cardiology	414	2.66	Q3
5	Circulation	409	34.39	Q1
6	International Journal of Cardiology	347	3.34	Q2
7	American Heart Journal	318	4.62	Q2
8	Medicine	318	1.51	Q3
9	Chest	240	8.70	Q1
10	European Journal of Cardio-thoracic Surgery	240	3.96	Q1

### 3.7. Analysis of reference co-occurrence and burst references

A total of 82 references were obtained. The top 10 cited references were Simpson L (2008), Uzun O (2007), Kirkpatrick J (2004), Lee VH (2007), Hoffmeier A (2014), Veugelers K (2004), Shanmugam G (2006), Gulati S (2004), Ekinci E (2004), and Loong F (2010) (Table 6).

**Table 6:** Top 10 references by citations.

Title	Journals	Authors	Year	Citations
Malignant primary cardiac tumors: review of a single institution experience	Cancer	Simpson L	2008	159
Cardiac tumours in children	Orphanet Journal of Rare Diseases	Uzun O	2007	154
Differential diagnosis of cardiac masses using contrast echocardiographic Perfusion Imaging	Cardiac Imaging	Kirkpatrick J	2004	149
Central nervous system manifestations of cardiac myxoma	Archives of neurology	Lee VH	2007	133
Cardiac tumors–diagnosis and surgical treatment	Deutsches Arzteblatt International	Hoffmeier A	2014	125
The granzyme B-serglycin complex from cytotoxic granules requires dynamin for endocytosis	Blood	Veugelers K	2004	118
Primary cardiac sarcoma	European Journal of Cardio-thoracic Surgery	Shanmugam G	2006	102
Central core disease	Indian Journal of Pediatrics	Gulati S	2004	94
Neurological manifestations of cardiac myxoma: a review of the literature and report of cases	Internal Medicine Journal	Ekinci E	2004	87
Diffuse large B-cell lymphoma associated with chronic inflammation as an incidental finding and new clinical scenarios	Modern Pathology	Loong F	2010	72

**Figure 11:**
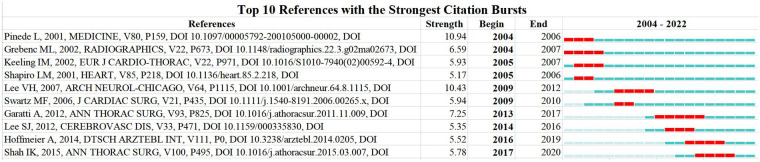
References burst analysis by CiteSpace.

In addition, a total of 10 references had the strongest citation bursts. From 2004 to 2007, the highlighted references were Pinede L (2001), Grenbenc ML (2002), Keeling IM (2002), and Shapiro LM (2001). From 2009 to 2012, the highlighted references were Lee VH (2007) and Swartz MF (2006). From 2013 to 2017, the highlighted references were Garatti A (2012), Lee SJ (2012), Hoffmeier A (2014), and Shah IK (2014) (Figure 11).

## 4. Discussion

Using the CiteSpace and VOSviewer software, this study conducted an in-depth analysis of the research status of atrial myxoma and summarized the knowledge base and current research hotspots of atrial myxoma. This bibliometric analysis revealed that the main research topics and hotspots in atrial myxoma included surgical methods, case reports, genetic and molecular studies. The results provide reference for future research on atrial myxoma and identify the potential direction of exploration.

### 4.1. Scientific co-operation among countries, institutions, and authors

In the national co-operation relationship map (Figure 2), the USA has the largest node diameter, followed by China, Turkey, Germany, United Kingdom, and India. The total number of papers issued by the above six countries was 557, accounting for 62% of the total number of papers issued by all countries. This indicates that the above countries have formed a strong co-operation network and have made great contributions to the atrial myxoma research field. The number of studies and depth of research by American scholars far exceed those in other countries.

From the perspective of co-operation between authors and institutions, as shown in Figure 4, a total of 23 scientific research institutions have studied atrial myxoma from different aspects, forming a co-operation network centered on the MayoClinic, Fujian Medical University, Zhejiang University, All India Institute of Medical Sciences, and Sichuan University. These five institutions have issued 54 papers, accounting for 6% of the total number of papers issued by all institutions. Fujian Medical University, All India Institute of Medical Sciences, and Sichuan University have conducted in-depth research; however, they are relatively isolated and have little co-operation with other institutions. The number of papers issued by the MayoClinic (*n* = 15) is higher than that of other institutions, accounting for 28% of papers issued by the above institutions. Its research focuses on surgical methods for atrial myxoma resection (22), neuroimaging (23), multimodal imaging, and 3D printing for the evaluation of tumor morphology (24, 25).

### 4.2. Co-citation among authors, journals, and cited documents

In the co-citation analysis of authors (Figure 5D), Reynenk K, Pinede L, Carney JA, Burke AP, and Stratakis CA are the top five authors regarding the number of cited authors, all of whom are foreign authors.

Among tumors with unknown biological behavior, Reynenk K was cited 312 times, indicating that his research contents had a high influence. The co-citation map of journals (Figure 10) shows that manuscript in Annals in Thoracic Surgery were cited most frequently, up to 1,067 times, indicating that manuscripts represented by Annals in Thoracic Surgery are the main source of literature research in the field of atrial myxoma. The literature with the highest citation rate is “Reynen K. Cardiac Myxomas. New England Journal of Medicine, 1995”, a review of cardiac myxomas, which summarizes the basic knowledge of cardiac myxomas from epidemiology, pathology, clinical manifestations, physical examination, diagnostic testing, differential diagnosis, treatment, etc. Although cardiac myxoma is histologically benign, it may have fatal effects due to its location. Different symptoms depend on the size, activity, and location of the tumor. Echocardiography, including the transesophageal approach, is the most important diagnostic tool; computed tomography (CT) and magnetic resonance imaging (MRI) may also be helpful. The tumor should be resected as soon as possible, which is associated with a good long-term prognosis and rare recurrence rate. In the follow-up examination, echocardiography is essential.

### 4.3. Research hotspots, trends, and prospects for atrial myxoma

#### 4.3.1. Research hotspots

Keywords are the central argument of a document; they reflect the core content of the document. Through keyword analysis, we can judge the research hotspots of atrial myxoma and determine the correct research direction. The keyword co-occurrence analysis map (Figure 6) has a large diameter, and the top keywords were as follows: cardiac myxoma, myxoma, tumor, experience, left atrial-myxoma, atrial-myxoma, diagnosis, atrial myxoma, echocardiography, heart, management, cardiac tumor, embolism, stroke, manifestation, and resection. The high frequency keywords in the above literature are closely related to the diagnosis and treatment of atrial myxoma and its complications. Cardiac myxoma is the most common primary benign cardiac tumor (accounting for 50% of primary cardiac tumors). The application of modern imaging technology in clinical practice, especially two-dimensional echocardiography, CT, and MRI, is helpful in the timely diagnosis of myxoma. However, the lack of specific clinical features, insufficient understanding of the disease, and rare prevalence may lead to misdiagnoses (26). Early diagnosis and treatment of atrial myxoma is very important to prevent embolism. The irregular surface of myxoma appears to be related to the embolic event (27).

Emergency physicians should evaluate the source of emboli when considering cerebrovascular accidents in patients under 35 years of age. A 28-year-old male with no medical history went to the emergency department for treatment because he began to develop acute left hemiplegia and facial ptosis on the day before arrival. Brain CT revealed focal ischemia; therefore, the emergency doctor performed bedside ultrasound and found a large, movable left atrial mass. After admission and confirmation of imaging, the mass was removed surgically. In otherwise healthy young people, a heart mass should be considered as the cause of unexplained stroke-like symptoms (28). It is reported that as many as 40% of patients with left atrial myxoma have systemic embolism, and half of them have brain involvement. The clinicopathological features of intracerebral embolism in left atrial myxoma and its association with the progressive implantation of tumor emboli and the potential pathogenic effects of interleukin-6 and matric metalloproteinases have been reviewed previously (29).

The keyword clustering map (Figure 8) revealed that #1 atrial myxoma, #2 cardiac myxoma, #3 papillary fibroelastoma, #7 cardiac tumors, and #9 primary cardiac tumors are related to tumor types. Some researchers have proposed a hypothesis regarding the origin of cardiac myxoma, which includes evaluating the significance of the atrial septal vascular tangle in the development of cardiac myxoma. Cardiac myxoma is thought to originate from pluripotent cardiac stem cells, which are often attached to the left side of the atrial septum. One of the characteristics of the attachment site is the aggregation of thick-walled vessels, which has been observed before the development of myxoma (30). A previous study compared the histology of a normal atrial septum and a septal flap resected with myxoma to evaluate the significance of the “vascular tangle” in the tumorigenesis of atrial myxoma. Based on the research results, a two-step hypothesis was proposed: the first step was to stimulate pluripotent cells to differentiate into endothelium and smooth muscle cells in the process of angiogenesis, and the subsequent step was the production of rich mucopolysaccharides that separated the smooth muscle cells, which would explain the rings, bands, or nests of myxoma cells around the endothelial lining space (31).

Regarding the keyword “intracranial aneurysm”, an analysis conducted by Yuan SM et al. revealed the advantages of multiple fusiform aneurysms of the middle cerebral artery associated with cardiac myxomas. Brain aneurysms occurred 53 months after cardiac myxoma resection. Left atrial myxomas are the most common cause of cerebral aneurysms. Removal of cardiac myxoma cannot avoid the occurrence of cerebral aneurysms; therefore, brain imaging monitoring is recommended even after cardiac myxoma resection (32). The neuroimaging manifestations of intracranial lesions related to myxoma vary, including multiple cerebral infarctions, aneurysm formation, intracranial focal hemorrhage, and space occupying lesions. Assessing the long-term progress in patients within 2 years of primary tumor resection requires regular MRI and CT angiography/magnetic resonance angiography/digital subtraction angiography. Furthermore, stable and effective chemotherapy drugs are urgently needed (33).

Regarding the keyword “complications”, myxoma can cause embolism, obstruction, and heart failure; however, it rarely causes chylothorax. Liao Z et al. reported a case of chylothorax caused by left atrial myxoma, which responded to diuretic treatment and was subsequently cured by resection of the cardiac myxoma (34). An article on the characteristics of ischemic stroke complications of cardiac myxoma patients in a single institution in Eastern China reviewed the medical records of 160 cardiac myxoma patients from January 2006 to December 2019. They were divided into a stroke group or non-stroke group. The study identified the three most common neurological symptoms (hypoesthesia, hemiplegia, and facial paralysis), and that involvement of the middle cerebral artery and multiple lesions are clear signs of stroke in cardiac myxoma patients. Surgical resection is a relatively safe operation for the treatment of both stroke and non-stroke patients (35).

“Transesophageal echocardiography” (TEE) can usually provide sufficient information for surgical resection. TEE with high sensitivity provides an excellent morphological definition that is helpful for diagnosis and follow-up (36, 37). TEE is important and practical for patients undergoing intracardiac mass resection during the perioperative period, and is helpful for monitoring and guidance during the surgical process (38, 39).

“Carney syndrome” can be divided into sporadic and familial; it is a rare autosomal dominant genetic disease and the most common familial cardiac myxoma. Most patients have an inactivation mutation of *PRKAR1A* on chromosome 17q22-24. Patients with Carney syndrome may have general symptoms of cardiac myxoma, such as fatigue, fever, body mass decline, myalgia, arthralgia, etc. In addition, they may also have skin lesions, lentigo, skin myxoma, and other characteristic skin manifestations, as well as multiple adenomas in the whole body (40, 41). If cardiac myxomas occur frequently at a relatively young age, the possibility of Carney syndrome should be considered (42).

“Minimal access” is associated with the development of surgical methods for myxoma removal. The “Epstein–Barr virus” (EBV) and “actin isoforms” are associated with the pathology of atrial myxoma. Jantuan E et al. suggested that cardiac myxoma can upregulate autophagy and create a favorable environment for EBV-driven tumorigenesis. This study is the first to describe the expression of autophagy-related genes and proteins in the tumor microenvironment in cardiac myxoma. The microenvironment of cardiac myxoma is dynamic; there are many cell types and different molecular pathways at work (43). Surgical resection of cardiac myxoma is still the main treatment method to avoid systemic or pulmonary embolism and harmful complications. However, recurrence is not uncommon and may be related to incomplete surgical resection (e.g., leaving some myxoma cells in the atrial septum) and/or that only some specific genetic characteristics are known (e.g., Carney complex). In the near future, the proposed miRNA-based therapy may play a key role in avoiding recurrence. Although the pathological groundbreaking research is promising, further research is still needed to understand how miRNA signal transduction directly regulates the pathophysiology of cardiac tumors. Further research should aim to investigate the causal relationship between different miRNAs and cell overgrowth, considering myxoma and other histological types of cardiac tumors. Thus, miRNA-based treatment of cardiac myxoma is a new potential therapeutic application (18).

The keywords burst analysis (Figure 9) shows the transfer of research hotspots in different periods and judges the potential development trend and frontier research. From 2008 to 2012, while focusing on disease diagnosis, surgical resection, as the most effective method to treat atrial myxoma (44), was a research hotspot. From 2013 to 2017, we gradually paid more attention to the role of follow-up (45) on patients after surgery. For patients with atrial myxoma, long-term follow-up should be carried out even after complete surgical resection, especially for patients with central nervous system manifestations before resection of atrial myxoma. In addition, complications such as embolism and stroke have become the focus of attention, and surgical treatment remains a high concern.

From 2018 to 2022, medical reports have attracted more and more attention and have become a hotspot. In addition, the risk factors of embolism in atrial myxoma patients has been gained increasing research attention. NYHA class (I/II), hypertension, irregular tumor surface, atypical tumor location, tumor basal stenosis, and elevated fibrinogen are important risk factors for embolism in cardiac myxoma patients; for such patients, early surgery may help to prevent embolism (46). Atrial fibrillation, irregular tumor surface, increased tumor size, and increased left atrial diameter were associated with an increased risk of embolism in patients with left atrial myxoma. Due to the increased possibility of embolism, early surgery should be arranged for such patients (47).

The surgical treatment of cardiac myxoma is also constantly improving. Structural cardiac abnormalities, such as atrial myxoma and patent foramen ovale, may play a role in brain injury. Because they can lead to abnormal cerebral embolism and cardiomyopathy, they may be related to sudden cardiogenic embolism events, and are increasingly valued (48).

In recent years, increasing attention has been paid to case reports, which promote our understanding of atrial myxoma in many ways. Kynta R reported a patient with chronic liver disease; a subsequent investigation found that there were masses in his right atrium and ventricle, which were consistent with heart tumors. During the operation, a huge mass was removed from the right atrium. The tumor stem originated from the tympanic pharyngeal flap. The histological results are consistent with myxoma. This is a case of Budd–Chiari syndrome caused by right atrial myxoma, which is extremely rare (49). Ertürk T et al. reported a case of giant left atrial myxoma with cough syncope syndrome (50). Cardiac myxoma mainly originating from the left atrium is the most common type of benign cardiac tumor, whereas biatrial myxoma is extremely rare. Fan C et al. reported a rare case of exertional dyspnea and intermittent chest discomfort in a 55-year-old man due to a huge bilateral atrial mass with atrial fibrillation and hepatic hemangioma (51).

Baugh et al. described a rare case of a 62-year-old male with incidental fibroin associated diffuse large B-cell lymphoma (FA-DLBCL) and atrial myxoma. The general immunophenotypical characteristics of the entity are an activated B cell phenotype and EBV-positive; however, this case was a germinal center B cell phenotype and EBV-negative. The article suggests expanding the definition of FA-DLBCL to include EBV-negative cases (52). The most common neurological complication associated with atrial myxoma is cerebral infarction caused by embolism. Early complete resection of giant cardiac myxoma is the key to the treatment and prevention of stroke recurrence. Chang W et al. reported a successful case in which thrombolysis and arterial embolus removal were used as bridging therapy for early complete resection of giant cardiac myxoma for stroke treatment and prevention of recurrence (53). A 63-year-old man was admitted to hospital due to acute left heart failure after doing farm work. Bedside echocardiography found a huge left atrial myxoma and he rapidly developed into refractory cardiogenic shock. Extracorporeal membrane oxygenation (ECMO) of venous arteries and veins was immediately performed and the patient was transferred for further surgery, with good results. Therefore, it is recommended to conduct echocardiographic evaluation and surgical resection of myxoma in a timely manner. ECMO can serve as a bridge between metastasis and the perioperative period (54). Ma S et al. reported a case of missed diagnosis of right atrial myxoma; digestive symptoms, systemic symptoms, and immune system disorders occurred, leading to tricuspid valve obstruction. This case reported the critical result of a missed diagnosis of right atrial myxoma, and indicated the importance of systematic examination of patients. In addition, it calls for early diagnosis of cardiac myxoma and consideration of drug targets to inhibit the development of cardiac myxoma (55).

Surgical treatment of atrial myxoma is being increasingly abundant and includes right chest minimally invasive small incision surgery, thoracoscopic surgery, robot surgery, etc. (12–16). With the continuous development and improvement of new instruments and technologies, the surgical treatment of atrial myxoma can be improved further. With the application of rapid rehabilitation surgery in cardiac surgery, cardiac myxoma resection is expected to enter the field of daytime surgery, and the technology in minimally invasive cardiac surgery will have a broader application prospect.

#### 4.3.2. Trends and prospects

In addition to the basic knowledge about the research topic, we also generalized the research hotspots in the past years and speculated frontiers in the near future. In the first frontier, surgical methods, the research gap is to explore safe, convenient, efficient and rapidly popularized surgical methods combined with new instruments and technologies. As for the second frontier, case report, the research gap is to provide more case reports, widely accumulate experience, and explore summaries from individual cases to commonalities. As for the third frontier, genetic and molecular studies, The research gap is to understand myxoma at the genetic and molecular levels, clarify its pathogenesis, and develop targeted drugs. These research gaps can guide future researchers to decipher the puzzles and improve their cooperation. Health policies can be supplemented in the future.

### 4.4. Limitations

There are still some limitations to our study: (1) Recently published articles may have low citations due to the limited time available for citations, and thus the study may be prone to research bias. (2) This study only included articles and reviews published in English, which may have overlooked some of the literature. (3) Only the citations and abstracts of the literature were analyzed and may have missed some essential information in the main text.

### 4.5. Future directions

Regarding the limitations mentioned above, our future research should: (1) With the rapid development of big data, this study may have a short timeframe and needs to be updated regularly. (2) The possible minor subtopics of atrial myxoma should be classified for more detailed statistical analysis. (3) When bibliometrics technology becomes more advanced, non-English sources will also be included in the study to obtain more comprehensive and accurate results.

## 5. Conclusions

In this study, CiteSpace software was used to analyze the research literature in the field of atrial myxoma over the past 20 years. This bibliometric analysis revealed that the main research topics and hotspots in atrial myxoma included surgical methods, case reports, genetic and molecular studies.

## Data Availability

The raw data supporting the conclusions of this article will be made available by the authors, without undue reservation.

## References

[1] Burke A Tavora F. The 2015 WHO classification of tumors of the heart and pericardium. J Thorac Oncol. (2016) 11(4):441–52. 10.1016/j.jtho.2015.11.009 26725181

[2] Ceresa F Calarco G Franzì E Patanè F. Right atrial lipoma in patient with cowden syndrome. Interact Cardiovasc Thorac Surg. (2010) 11(6):803–4. 10.1510/icvts.2010.245001 20852328

[3] Ceresa F Sansone F Rinaldi M Patanè F. Left atrial paraganglioma: diagnosis and surgical management. Interact Cardiovasc Thorac Surg. (2010) 10(6):1047–8. 10.1510/icvts.2009.231043 20197349

[4] MacGowan SW Sidhu P Aherne T Luke D Wood AE Neligan MC Atrial myxoma: national incidence, diagnosis and surgical management. Ir J Med Sci. (1993) 162(6):223–6. 10.1007/BF02945200 8407260

[5] Kunioka S Fujita K Iwasa S Murakami H Kamiya H Yamazaki K A rare form of cardiac myxoma: interatrial septum tumor. J Surg Case Rep. (2020) 2020(9):rjaa333. 10.1093/jscr/rjaa333 32968478 PMC7497063

[6] Kearney A Corry N Menown IBA. Massive left atrial myxoma presenting with troponin-positive chest pain. Cardiol Ther. (2020) 9(2):577–80. 10.1007/s40119-020-00187-2 32720069 PMC7584706

[7] Yoon DH Roberts W. Sex distribution in cardiac myxomas. Am J Cardiol. (2002) 90(5):563–5. 10.1016/s0002-9149(02)02540-7 12208428

[8] Keeling IM Oberwalder P Anelli-Monti M Schuchlenz H Demel U Tilz GP Cardiac myxomas: 24 years of experience in 49 patients. Eur J Cardiothorac Surg. (2002) 22(6):971–7. 10.1016/s1010-7940(02)00592-4 12467822

[9] Orlandi A Ciucci A Ferlosio A Genta R Spagnoli LG Gabbiani G. Cardiac myxoma cells exhibit embryonic endocardial stem cell features. J Pathol. (2006) 209(2):231–9. 10.1002/path.1959 16508920

[10] Kuyama N Hamatani Y Fukushima S Ikeda Y Nakai E Okada A Left ventricular myxoma with carney complex. ESC Heart Fail. (2018) 5(4):713–5. 10.1002/ehf2.12282 29542870 PMC6073037

[11] Shrestha S Raut A Jayswal A Yadav RS Poudel CM. Atrial myxoma with cerebellar signs: a case report. J Med Case Rep. (2020) 14(1):29. 10.1186/s13256-020-2356-5 32051024 PMC7017557

[12] Kadiroğulları E Onan B Aydın Ü Başgöze S Şen O. A comparison of robotically-assisted endoscopic versus sternotomy approach for myxoma excision: a single-center experience. Turk Gogus Kalp Damar Cerrahisi Derg. (2020) 28(3):450–9. 10.5606/tgkdc.dergisi.2020.19278 32953207 PMC7493598

[13] Ko PJ Chang CH Lin PJ Chu JJ Tsai FC Hsueh C Video-assisted minimal access in excision of left atrial myxoma. Ann Thorac Surg. (1998) 66(4):1301–5. 10.1016/s0003-4975(98)00759-0 9800824

[14] Santana O Reyna J Grana R Buendia M Lamas GA Lamelas J. Outcomes of minimally invasive valve surgery versus standard sternotomy in obese patients undergoing isolated valve surgery. Ann Thorac Surg. (2011) 91(2):406–10. 10.1016/j.athoracsur.2010.09.039 21256280

[15] Shi J Wang Y Wang Q Bing X Ma Z. Simultaneously performed, totally endoscopic left atrial myxoma resection and lobectomy. Heart Surg Forum. (2020) 23(3):E292–4. 10.1532/hsf.2839 32524982

[16] Doulamis IP Spartalis E Machairas N Schizas D Patsouras D Spartalis M The role of robotics in cardiac surgery: a systematic review. J Robot Surg. (2019) 13(1):41–52. 10.1007/s11701-018-0875-5 30255360

[17] Cheng N Wu Y Zhang H Guo Y Cui H Wei S Identify the critical protein-coding genes and long noncoding RNAs in cardiac myxoma. J Cell Biochem. (2019) 120(8):13441–52. 10.1002/jcb.28618 30912168

[18] Nenna A Loreni F Giacinto O Chello C Nappi P Chello M miRNAs in cardiac myxoma: new pathologic findings for potential therapeutic opportunities. Int J Mol Sci. (2022) 23(6):3309. 10.3390/ijms23063309 35328730 PMC8954653

[19] Zhu S Li L Gu Z Chen C Zhao Y. 15 years of small: research trends in nanosafety. Small. (2020) 16(36):e2000980. 10.1002/smll.202000980 32338444

[20] Chen C. Searching for intellectual turning points: progressive knowledge domain visualization. Proc Natl Acad Sci U S A. (2004) 101 (Suppl 1):5303–10. 10.1073/pnas.0307513100 14724295 PMC387312

[21] Ma D Guan B Song L Liu Q Fan Y Zhao L A bibliometric analysis of exosomes in cardiovascular diseases from 2001 to 2021. Front Cardiovasc Med. (2021) 8:734514. 10.3389/fcvm.2021.734514 34513962 PMC8424118

[22] Konecny T Reeder G Noseworthy PA Konecny D Carney JA Asirvatham SJ. Percutaneous ablation and retrieval of a right atrial myxoma. Heart Lung Circ. (2014) 23(11):e244–7. 10.1016/j.hlc.2014.07.059 25240574

[23] Brinjikji W Morris JM Brown RD Thielen KR Wald JT Giannini C Neuroimaging findings in cardiac myxoma patients: a single-center case series of 47 patients. Cerebrovasc Dis. (2015) 40(1–2):35–44. 10.1159/000381833 26068450

[24] Ali M Pham AN Pooley RA Rojas CA Mergo PJ Pham SM. Three-dimensional printing facilitates surgical planning for resection of an atypical cardiac myxoma. J Card Surg. (2020) 35(10):2863–5. 10.1111/jocs.14896 32720392

[25] Saef J Jellis C Unai S Tan C Dresing T Ayoub C. Multimodality imaging evaluation of incidentally discovered intracardiac mass. Echocardiography. (2022) 39(6):837–40. 10.1111/echo.15354 35505607

[26] Fomin VV Kogan EA Morozova NS Chichkova NV Komarov RN Kurasov NO Cardiac myxoma: challenge in diagnostics. Case report. Ter Arkh. (2021) 93(4):470–7. 10.26442/00403660.2021.4.200685 36286783

[27] Wen XY Chen YM Yu LL Wang SR Zheng HB Chen ZB Neurological manifestationes of atrial myxoma: a retrospective analysis. Oncol Lett. (2018) 16(4):4635–9. 10.3892/ol.2018.9218 30214598 PMC6126161

[28] Bradshaw JC Arthur J Lewis ZB. Ultrasound-assisted diagnosis of embolic cerebrovascular accident from left atrial myxoma in the emergency department. J Emerg Med. (2021) 61(4):e60–3. 10.1016/j.jemermed.2021.05.010 34210532

[29] Aguilar C Carbajal T Beltran BE Segura P Muhammad S Choque-Velasquez J. Cerebral embolization associated with parenchymal seeding of the left atrial myxoma: potential role of interleukin-6 and matrix metalloproteinases. Neuropathology. (2021) 41(1):49–57. 10.1111/neup.12697 32776398

[30] Dübel HP Knebel F Gliech V Konertz W Rutsch W Baumann G Atypical vessels as an early sign of intracardiac myxoma? Cardiovasc Ultrasound. (2004) 2:13. 10.1186/1476-7120-2-13 15310408 PMC514718

[31] Vaideeswar P Yadav S. Vascular tangle in the inter-atrial septum—is it the source of cardiac myxoma? Indian J Pathol Microbiol. (2021) 64(3):469–71. 10.4103/IJPM.IJPM_1215_20 34341255

[32] Yuan SM. Cerebral aneurysms due to cardiac myxoma. J Coll Physicians Surg Pak. (2019) 29(8):763–7. 10.29271/jcpsp.2019.08.763 31358100

[33] Zhang S Zhang Q Yu H Liu L Sun R Song X Neuroimaging characteristics and long-term prognosis of myxoma-related intracranial diseases. Neuroradiology. (2020) 62(3):307–17. 10.1007/s00234-019-02314-w 31713666

[34] Liao Z Huang W Hu Q Wang Z Pan L Zheng W. Chylothorax caused by left atrial myxoma: a rare case report. J Card Surg. (2021) 36(12):4792–5. 10.1111/jocs.16074 34647368

[35] Zhang Y Ye Z Fu Y Zhang Z Ye Q Chen F Characterizations of ischemic stroke complications in cardiac myxoma patients at a single institution in eastern China. Neuropsychiatr Dis Treat. (2021) 17:33–40. 10.2147/NDT.S280641 33442253 PMC7800702

[36] Meng Q Lai H Lima J Tong W Qian Y Lai S. Echocardiographic and pathologic characteristics of primary cardiac tumors: a study of 149 cases. Int J Cardiol. (2002) 84(1):69–75. 10.1016/s0167-5273(02)00136-5 12104067

[37] Oliveira R Branco L Galrinho A Abreu A Abreu J Fiarresga A Cardiac myxoma: a 13-year experience in echocardiographic diagnosis. Rev Port Cardiol. (2010) 29(7–8):1087–100. 21066964

[38] Kumar US D Wali M Shetty SP Sujay KR. Left atrial myxoma—a tumor in transit. Ann Card Anaesth. (2019) 22(4):432–4. 10.4103/aca.ACA_232_18 31621681 PMC6813701

[39] Munirathinam GK Kumar B Singh H. Role of transesophageal echocardiography in the recurrent biatrial myxoma of uncommon origin. Ann Card Anaesth. (2022) 25(1):85–8. 10.4103/aca.ACA_71_20 35075027 PMC8865343

[40] Yuan SM Yan SL Wu N. Unusual aspects of cardiac myxoma. Anatol J Cardiol. (2017) 17(3):241–7. 10.14744/AnatolJCardiol.2017.7557 28321109 PMC5864986

[41] Correa R Salpea P Stratakis CA. Carney complex: an update. Eur J Endocrinol. (2015) 173(4):M85–97. 10.1530/EJE-15-0209 26130139 PMC4553126

[42] Yokoyama S Nagao K Higashida A Aoki M Yamashita S Fukuda N Diagnosis of carney complex following multiple recurrent cardiac myxomas. Gen Thorac Cardiovasc Surg. (2022) 70(1):87–91. 10.1007/s11748-021-01719-w 34642893 PMC8732819

[43] Jantuan E Chiu B Chiu B Shen F Oudit GY Sergi C. The tumor microenvironment may trigger lymphoproliferation in cardiac myxoma. Transl Oncol. (2021) 14(1):100911. 10.1016/j.tranon.2020.100911 33129111 PMC7586245

[44] Kuroczyński W Peivandi AA Ewald P Pruefer D Heinemann M Vahl CF. Cardiac myxomas: short- and long-term follow-up. Cardiol J. (2009) 16(5):447–54. 19753524

[45] Wan Y Du H Zhang L Guo S Xu L Li Y Multiple cerebral metastases and metastatic aneurysms in patients with left atrial myxoma: a case report. BMC Neurol. (2019) 19(1):249. 10.1186/s12883-019-1474-4 31646971 PMC6813067

[46] Liu Y Wang J Guo L Ping L. Risk factors of embolism for the cardiac myxoma patients: a systematic review and metanalysis. BMC Cardiovasc Disord. (2020) 20(1):348. 10.1186/s12872-020-01631-w 32711463 PMC7382866

[47] Kalçık M Bayam E Güner A Küp A Kalkan S Yesin M Evaluation of the potential predictors of embolism in patients with left atrial myxoma. Echocardiography. (2019) 36(5):837–43. 10.1111/echo.14331 30934139

[48] Kelley RE Kelley BP. Heart-brain relationship in stroke. Biomedicines. (2021) 9(12):1835. 10.3390/biomedicines9121835 34944651 PMC8698726

[49] Kynta RL Rawat S Lyngdoh BS Gunasekaran AK Fanai V Kapoor M Eustachian valve myxoma: a rare cause of budd-chiari syndrome. Gen Thorac Cardiovasc Surg. (2021) 69(8):1243–6. 10.1007/s11748-021-01641-1 34036487

[50] Tekin E E. Giant left atrial myxoma presenting with cough-syncope syndrome. Heart Surg Forum. (2021) 24(4):E7090–712. 10.1532/hsf.3991 34473020

[51] Fan C Zhang H Zhuang H Jiang Z Tan H Iroegbu CD Case report: giant biatrial myxoma mimicking malignant cardiac tumor in a patient with a hepatic angiomatous mass. Front Cardiovasc Med. (2021) 8:676807. 10.3389/fcvm.2021.676807 34124204 PMC8192690

[52] Baugh L Brown N Song JY Pandya S Montoya V Fibrin-Associated PA. EBV-negative diffuse large B-cell lymphoma arising in atrial myxoma: expanding the Spectrum of the entity. Int J Surg Pathol. (2022) 30(1):39–45. 10.1177/10668969211014959 33913371

[53] Chang WS Li N Liu H Yin JJ Zhang HQ. Thrombolysis and embolectomy in treatment of acute stroke as a bridge to open-heart resection of giant cardiac myxoma: a case report. World J Clin Cases. (2021) 9(25):7572–8. 10.12998/wjcc.v9.i25.7572 34616828 PMC8464453

[54] Tian L Zhang S Xu J Han X. Extracorporeal membrane oxygenation as a bridge between transfer and perioperative periods in refractory cardiogenic shock secondary to a large left atrial myxoma. Heart Surg Forum. (2021) 24(2):E215–6. 10.1532/hsf.3501 33798058

[55] Ma S Xu Q Shi R Zhang X Chen X. The omitted symptoms challenge the diagnosis of right atrial myxoma: a case report. BMC Cardiovasc Disord. (2020) 20(1):149. 10.1186/s12872-020-01413-4 32213175 PMC7093949

